# Epidemiology and factors influencing visual outcome in paediatric ocular trauma at a tertiary health institution in Ghana

**DOI:** 10.4314/gmj.v59i4.2

**Published:** 2025-12

**Authors:** Vera M Beyuo, Imoro Z Braimah, Benjamin Abaidoo, Akorfah Lassey, Vera A Essuman

**Affiliations:** 1 Lions International Eye Centre, Korle Bu Teaching Hospital, Accra, Ghana; 2 Ophthalmology Unit, Department of Surgery, University of Ghana Medical School, Accra, Ghana

**Keywords:** Ocular trauma, Paediatric, open globe injury, POTS, visual outcome

## Abstract

**Objectives:**

To describe the epidemiology of paediatric ocular trauma (POT) presenting at a tertiary health institution in Ghana and factors influencing visual outcome

**Design:**

Prospective study

**Setting:**

Korle-Bu Teaching Hospital, Accra, Ghana

**Participants:**

Children aged 0-15 years presenting with trauma involving the globe and/or adnexa from January 2022 to February 2023.

**Interventions:**

Clinical management of ocular trauma

**Main outcome measures:**

best-corrected visual acuity at 6 weeks, and factors influencing visual outcome

**Results:**

A significant proportion of children (59.2%) presenting with trauma were between ages 6-10 years (mean age 7.2±3.3 years), with the majority being male (70%). There was no caregiver at the time of injury in most cases (76%). Globe injuries were predominant (78.6%), and most (56.3%) were open globe injuries (OGIs). OGIs and organic objects as causes of injury were associated with unfavourable visual outcomes (p < 0.05). Early presentation, an inorganic object as the cause of injury, a favourable presenting visual acuity, a closed-globe injury, and a higher Paediatric Ocular Trauma Score (POTS) were associated with a favourable visual outcome. A third of children were blind (best-corrected visual acuity < 3/60) in the affected eye at follow-up.

**Conclusion:**

Open globe injuries were the most common type of injury and often resulted in poor visual outcomes. Better public awareness, caregiver education, and adult supervision can help prevent these injuries.

**Funding:**

None declared

## Introduction

Ocular trauma refers to physical injury to the eye and its associated structures.^1^ It is a significant cause of unilateral and bilateral blindness in both adults and children.^1^–^3^ Globally, ocular trauma causes about 3.9 million cases of bilateral and over 18 million cases of unilateral vision loss, making it the leading cause of unilateral blindness.^3^,^4^ Although isolated ocular trauma is usually not life-threatening, the sufferers must live with the sequelae, which may include disfigurement or loss of vision. Vision loss usually results from direct and or indirect damage to vital structures in the eye, as well as visual deprivation and amblyopia. Ocular trauma has a greater impact on children because prolonged visual morbidity affects their psychosocial development, future career and social opportunities, and overall quality of life.^5^

Visual outcomes following ocular trauma may depend on several factors. These include factors related to the trauma itself, such as type and severity,^6^–^9^ pre-hospital factors, such as delay in seeking medical care, or in-hospital factors, such as delays with initiating intervention.^9^,^10^ In this manuscript, we present the epidemiology of paediatric ocular trauma presenting to the Lions International Eye Centre (LIEC), Korle Bu Teaching Hospital (KBTH) in Accra and factors influencing visual outcome.

## Methods

### Study design

This was a prospective study involving 103 children aged 0 -15 years who presented with ocular trauma at the Lions International Eye Centre, KBTH, Accra, Ghana, from January 2022 to February 2023.

### Study site

The Korle Bu Teaching Hospital in Accra, Ghana, is a leading tertiary eye facility and referral centre for the southern region. Its Lions International Eye Centre offers specialised eye care, treats various eye diseases from within and outside the sub-region, and serves as a regional centre for training ophthalmologists in West Africa.^10^

### Procedures

Children with ocular trauma to the globe or adnexa (eyelids, lacrimal apparatus, orbital structures) were included, excluding those with previous trauma to the same eye or prior visual impairment. Consent was obtained from parents and assent from children over 8; for illiterate guardians, forms were translated, and thumbprints were used as signatures. Participants were enrolled consecutively until the target sample size was reached.

### Sample size determination

The sample size was based on Madan et al.'s study, which found 63.9% of paediatric ocular trauma cases involved open globe injury; ultimately, 103 children were recruited..^11^

### Ethical clearance

Ethical clearance was obtained from the hospital's Institutional Review Board (Number KBTHIRB/00082/2021]. The study adhered to the Declaration of Helsinki on ethical principles for medical research involving human subjects.

A structured questionnaire was used in collecting demographic and clinical data. History of the cause of injury, place of injury, type/mechanism of injury, time of injury and pre-hospital treatment given were recorded. Visual acuity (VA) was assessed using an appropriate method for age.

The ocular adnexa were examined for lacerations, oedema, ecchymosis, and other abnormalities. The cornea and sclera were examined for lacerations, abrasions, ulcers and retained foreign bodies. When a laceration was present, the zone of laceration, the presence of leakage (using Siedel's test) and the presence of iris prolapse within the wound were noted. Presence of hyphaema and other anterior chamber abnormalities was noted. The lens was examined to assess for lens capsular rupture, lens dislocation, and/or the presence of a traumatic cataract.

The posterior segment of the affected eye was assessed using gentle ocular ultrasonography (B scan) when there was no visualisation of it. On subsequent visits, when the media were clear, fundoscopy was performed.

The Birmingham Eye Trauma Terminology (BETT) system was used to classify globe injuries as follows.^12^ Closed globe (contusion, lamellar laceration, superficial foreign body), open globe (laceration- subclassified as penetrating injury, perforating injury, an intraocular foreign body, and globe rupture). For open globe injuries, the paediatric ocular trauma score as described by Acar et al^13^ was used to assess injury severity and prognosis.

Presentation was considered early if it occurred within 48 hours of injury and delayed if it occurred after 48 hours. Injuries were categorised as organic (sticks, broomsticks, stones) or inorganic (metal, plastic, fight, play). Home or pre-hospital management was classified as orthodox (none, topical antibiotics, use of shield) or unorthodox (holy water, breast milk, face washing).

### Treatment

The study participants received treatment according to the type of injury, in accordance with standard protocols at the LIEC, and this was also documented in the questionnaire.

**Lacerations:** All corneal and corneoscleral lacerations were repaired on the day of presentation or within 24 hours of presentation. Repair was done under general anaesthesia (GA) using 10-0 Nylon suture. Postoperative medications included 1% Prednisolone acetate, 0.3% Ofloxacin, and 1% cyclopentolate eye drops. Lid lacerations were repaired under general anaesthesia using Vicryl 6-0 suture.

**Closed-globe injuries:** Hyphaema was managed conservatively or surgically, depending on severity. For small to moderate hyphaema, topical steroids, 1% Cyclopentolate and intraocular pressure (IOP) lowering drops were prescribed. Anterior chamber washout was performed early for large or total hyphaema, corneal staining, persistently high IOP, children with sickle cell disease, and non-clearing hyphaema, to reduce the risk of amblyopia.

**Traumatic cataract:** For children with traumatic cataract, in the presence of anterior capsular rupture, anterior chamber washout and lens aspiration were performed within 24 hours, and, if there was sufficient capsular support, a posterior chamber intraocular lens was implanted. Where anterior capsular support was thought to be inadequate, the patient was left aphakic for secondary IOL implantation. When the lens capsule was intact, patients were scheduled for paediatric clinic and lens aspiration, with possible posterior capsulotomy, anterior vitrectomy, and posterior chamber IOL implantation.

**Endophthalmitis:** Where this diagnosis was made, a vitreous tap for culture and sensitivity was taken, followed by intravitreal injection of Ceftazidime 2.25mg/0.1ml and Vancomycin 1mg/0.1ml under general anaesthesia.

**Traumatic corneal abrasions and ulcers:** These were managed with topical antibiotics. Corneal abrasions were managed with a combination of antibiotic drops (e.g., topical ciprofloxacin or ofloxacin) and ointment (e.g., Oxypol; Oxytetracycline 5 mg Polymyxin B sulfate 10,000 I.U. and tobramycin ointment).

Corneal ulcers were managed according to microscopy, culture, and sensitivity results, with broad-spectrum antibiotics such as fortified cefuroxime, fortified gentamicin, and gatifloxacin for bacterial ulcers, and antifungal eye drops such as natamycin for fungal infections. Most treatment was covered by the National Health Insurance Scheme (NHIS). Parents, guardians, or NGOs paid out-of-pocket for uncovered expenses.

Ocular examinations were done at presentation, post-intervention week 2 and post-intervention week 6, and findings were documented. For patients who had second operations or interventions, the final 6-week findings were documented after the last surgical intervention. In assessing visual outcomes, visual acuity was converted to LogMAR for analysis and categorised as normal, mild visual impairment (VI), moderate VI, severe VI, and blindness according to the WHO classification.^14^

Categorisation was done to facilitate analysis of data on factors affecting visual outcome. A favourable visual outcome was defined as visual acuity of better than 6/60 (LogMAR 1.0).^15^,^16^ An unfavourable visual outcome was defined as visual acuity of 6/60 (LogMAR 1.0) or worse.

Data were analysed using the Statistical Package for the Social Sciences (SPSS) version 25. Descriptive data were presented in frequency tables, with counts, means, and percentages. Differences in proportions of categorical variables were analysed using the Chi-square test. The correlation between paediatric ocular trauma scores and final visual outcome was analysed using Spearman's correlation coefficient. Univariate and multivariate logistic regression analyses were conducted to assess the association between post-intervention visual outcomes and variables such as age, presenting visual acuity, cause of injury, type of injury, and type of home management. The significance level was set at p < 0.05.

## Results

### Demographic characteristics of study participants

The mean age of the children was 7.2±3.3 years (ranged from 1.2 years to 15.0). More than half (n=61, 59.2%) of the children were 6-10 years, 72(70%) were males and 81(78.6%) were from urban areas.

### Place of injury

Most (n=68, 66.0%) of the children experienced trauma at home. Of these, 36 (52.9%) of the home injuries were among children aged 6-10 years. The majority (n=15, 65.2%) of injuries occurring at school were also among children aged 6-10 years. Other places where injuries occurred included farms (n=5, 4.9%), road traffic accidents (n=4, 3.8%), recreational parks (n=1, 1.0%), ways from school (n=1, 1.0%), and at parties (n=1, 1.0%). Only twenty-five (24.0%) caregivers were present at the time of trauma.

### Laterality of trauma among the children

All injuries were unilateral, with the right eye affected in 62 (60.2%) of the participants.

### Type and aetiology of ocular trauma/injury

**Figure 1:**
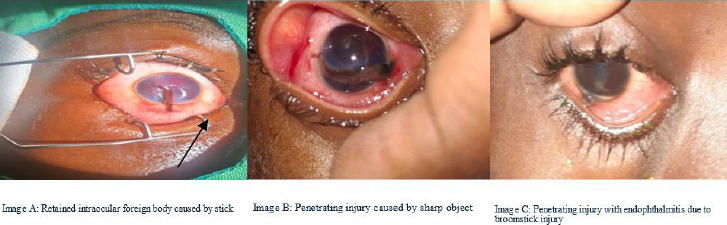
Penetrating injuries

**Figure 2:**
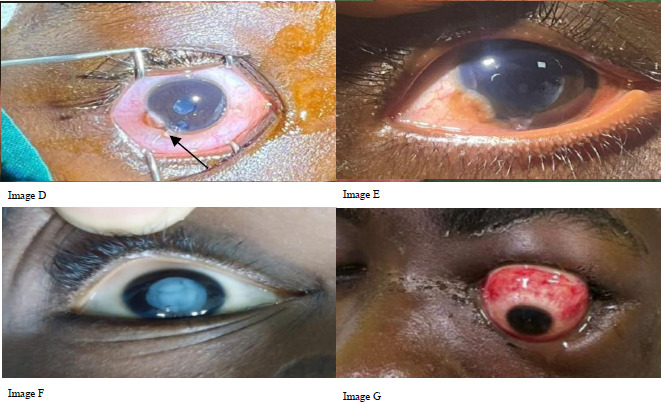
Perforating glob e injuries (Image D-Perforating globe injury with traumatic cataract and vitreous prolapse. Image E-Post laceration repair, lens aspiration and secondary intraocular lens. BCVA at 6 weeks after secondary IOL was 6/12. Image F-Traumatic cataract with rupture of anterior capsule. Image G-Globe subluxation following a fall in 15-year-old male.)

The top three causes of ocular trauma were sharp objects (31, 30.1%), sticks (21, 20.4%) and stones (12, 11.7%). Globe injury occurred in 81 (78.6%) of the cases.). More than half (58, 56.3%) of the injuries were OGIs. (Table 2). Under OGI, 51(88%) had lacerations and 7(12%) had globe rupture. Of the 51 who had lacerations, 41(80%) had penetrating injuries (Figure 1), 6(12%) had perforating injuries (Figure 2, image D), and 4(8%) had intraocular foreign body (IOFB). Of those with CGI, 90.4% were classified as contusions, 4.8% as lamellar lacerations, and 4.8% as superficial foreign bodies.

**Table 2:** Causes of trauma and type of trauma in children presenting with ocular trauma

Causes	Type of injury N (%)			Total N (%)	p-value

	Closed globe	Open globe	Lid laceration		
**Sharp object**	3(2.9)	19(18.4)	9(8.7)	31(30.1)	0.001
**Stick**	4(3.9)	17(16.5)	-	21(20.4)	
**Thrown stone**	2(1.9)	8(7.8)	2(1.9)	12(11.7)	
**Broomstick**	2(1.9)	8(7.8)	-	10(9.7)	
**Sports related**	3(2.9)	2(1.9)	3(2.9)	8(7.8)	
**Fist fight**	5(4.9)	1(1.0)	1(1.0)	7(6.8)	
**Road traffic accident**	2(1.9)	0(0.0)	4(3.9)	6(5.8)	
**Fall**	1(1.0)	1(1.0)	3(2.9)	5(4.9)	
**Others** **Total**	1(1.0) 23(22.3)	2(1.9) 58(56.3)	- 22(21.4)	3(2.9) 103(100.0)	

**Figure 3:**
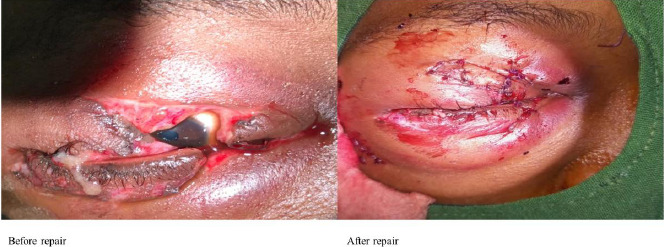
Multiple lid lacerations in young girl following Road Traffic Accident

The commonest presentation of CGI was traumatic cataract (Figure 2, image F). A total of 22 (21.4%) had adnexal/lid lacerations (Figure 3).

Others included 1(1.0%) each of PVC pipe injury, cork of wine (champagne), and metal railings.

### Home management of injury among the children with ocular trauma at the KBTH

Most (70, 68.0%) of the children did not receive any pre-hospital management at initial presentation. Twelve children used topical antibiotics at home when the injury occurred. Other forms of home management of injuries included washing with water (6, 5.8%), medications not prescribed from hospital (4, 3.9%), cold (ice) compress (4, 3.9%), dressing with gauze (2, 1.9%), use of breast milk (1, 1.0%), covering with bandage (1, 1.0%), holy water (1, 1.0%), removal of broomstick (1, 1.0%).

### Time interval between trauma and presentation

Table 3 provides the interval between trauma and reporting to the hospital.

**Table 3:** Time interval between trauma and presentation at the Korle Bu Teaching Hospital

Time	Frequency	Percentage (%)
**<24 hours**	40	38.8
**24 - <48 hours**	13	12.6
**2-6 days**	37	35.9
**1 week**	1	1.0
**2-4 weeks**	12	11.7

### Visual outcome in children with ocular trauma

The median VA at baseline was 2.6 LogMAR. The minimum and maximum VA were 0.00 LogMAR and 2.90 LogMAR, respectively. The median VA at 2 weeks was 0.7 (2.6) LogMAR. Minimum VA was 0.00 LogMAR, maximum VA was 2.90 LogMAR. The median VA at 6 weeks = 0.5 LogMAR (Minimum VA was 0.00 LogMAR, and maximum VA was 2.90 LogMAR). For all grades of vision (normal vision, mild VI, moderate VI, severe VI, and blind), there was improvement from baseline to 6 weeks of follow-up, which was statistically significant (p > 0.001). (Table 4).

**Table 4:** Visual outcome in children with ocular trauma at the Korle Bu Teaching Hospital

Visual acuity	Visual outcomes		P-value

	Baseline BCVA	6-weeks follow-up BCVA	
**Normal**	27(27.6)	46(47.4)	
**Mild VI**	1(1.0)	6(6.2)	
**Moderate VI**	11(11.2)	8(8.2)	
**Severe VI**	3(3.1)	1(1.0)	
**Blind**	56(57.1)	36(37.1)	0.001

### BCVA – Best corrected visual acuity

Using the Paediatric Ocular Trauma Score (POTS) for open-globe injury in children presenting with ocular trauma, 29 (50%) of children with open-globe injury had a POTS score of 1. The frequency of other POTS classes in open-globe injuries is presented in Table 5.

**Table 5:** Paediatric Ocular Trauma Score (POTS) for Open globe injury in children presenting with ocular trauma at Korle Bu Teaching Hospital

POTS	Open globe injury N (%)
**1**	29(50%)
**2**	14(24.1)
**3**	12(20.7)
**4**	3(5.2%)
**Total**	58(100%)

### Relationship between paediatric ocular trauma scores and final visual acuity

There was a strong negative correlation between POTS and final visual outcome (Spearman's correlation: r = -0.611; p-value = 0.001). Thus, the higher the POTS, the lower the LogMAR VA (better VA).

### Factors influencing visual outcomes post-ocular trauma in children

Univariate analysis showed that early presentation, favourable baseline visual acuity, CGI, and an inorganic object as the cause of injury were associated with favourable visual outcomes post-ocular trauma in children (p < 0.05). (Table 5). In a multivariate model, CGI and inorganic causes of injury were the only factors associated with favourable visual outcomes post-ocular trauma in children (p < 0.05). (Table 6).

**Table 6:** Univariate analysis of factors influencing visual outcomes post ocular trauma in children at the Korle Bu Teaching Hospital

Risk factor	Odds ratio	95% Confidence interval	P-value
**Age group**			
**<10 years**	0.4	0.1-1.2	0.099
**10-15 years**	Ref. cat.	-	
**Sex:**			
**Female**	1.1	0.4-2.5	0.895
**Male**	Ref. cat	-	
**Place of Injury:**			
**Home**	0.7	0.2-2.5	0.548
**School**	0.8	0.2-3.3	0.726
**Others**	Ref. cat		
**Presence of care-giver**	2.0	0.7-5.6	0.187
**Time of presentation**			
**Early** **Late**	3.2 Ref. cat	1.4-7.6 -	0.007*
**Home management**			
**Orthodox** **Non-orthodox**	1.1 Ref. cat	0.2-6.9	0.915
**Baseline VA**			
**Favourable** **Unfavourable**	2.9 Ref. cat	2.0-4.2 -	0.001*
**Type of injury:**			
**Closed** **Lid laceration** **Open**	11.1 3.6 Ref. cat	2.4-52.0 0.6-21.1 -	0.002* 0.160
**Cause of injury:**			
**Inorganic** **Organic**	2.3 Ref. Cat.	1.3-3.9 -	0.003*

* Significant values. Ref. cat. = reference category; VA= visual acuity

## Discussion

A significant proportion (59.2%) of children presenting with ocular trauma were aged 6-10 years, the majority being males. It was found that most injuries were globe injuries, particularly OGIs and occurred at home. Notably, there was no caregiver at the time of injury in most cases. Open globe injuries and organic object as cause of injury were associated with unfavourable visual outcomes (p< 0.05), while early presentation, inorganic object as cause of injury, favourable presenting visual acuity, closed globe injury, and higher POTS were associated with favourable visual outcomes.

The mean age of our cohort(7.2 years) is comparable to other studies from India^11^, Turkey^17^ and the United Kingdom (UK).^4^ Our finding also aligns with a study conducted in Kaduna, Nigeria by Kyari et al^18^ where the mean age was reported to be 7.73 years. This suggests a need for school eye health programmes to educate this age group on ocular trauma prevention. Most children with ocular trauma in our study were males, which is also consistent with several other studies.^4^,^12^,^17^–^20^ Li et al, in a 10-year retrospective study conducted in China on paediatric ocular trauma, reported that nearly three-quarters of their study participants were males.^21^ This is likely because boys are generally more active, aggressive and adventurous compared to their female counterparts.^22^,^23^ Some studies have attributed these differences to biological, psychosocial and environmental factors.^22^,^23^ Telford et al^23^ suggested that a lesser percentage of body fat, coupled with higher lean muscle weight, compared with their female contemporaries, could account for the male dominance in physical activities. Additionally, society expects girls to be calm and demure, while boys are expected to be strong and aggressive. Again, Telford et al^23^ mentioned that male children are more likely to be encouraged by their parents to engage in physical activity compared to female children.

Home was the commonest place of injury (66%). This is similar to what was found by Gatsey et al^1^ who reported that 70% of paediatric ocular trauma occurred at home, which is further corroborated by Serrano et al.^24^ In a Colombian study, he reported that the majority of paediatric ocular trauma occurred at home. In this current study, caregivers or adult supervisors were present in only a quarter of cases at the time of ocular trauma, which is consistent with what was reported by Grieshaber et al^25^ in a South African study on penetrating eye injuries in children. They reported that caregivers were absent in 85% of cases. In the UK, a retrospective study on paediatric ocular trauma by Sii et al^4^ suggested that 90% of cases of paediatric ocular trauma can be prevented if there is better supervision of children.

Studies on paediatric ocular trauma have demonstrated geographic variations in the causes of ocular injury.^4^,^16^,^26^–^29^ Sports-related ocular trauma occurs commonly in children from developed countries^4^,^16^ while children from developing countries and low socioeconomic status tend to sustain ocular trauma commonly from organic materials such as sticks, broomsticks and stones.^1^,^25^,^28^–^30^ Okoye et al found sticks and stones to be the leading cause of paediatric ocular trauma in their study involving 32 Nigerian children.^30^ In this study, sharp objects and organic objects like sticks and stones were the leading causes of ocular trauma in children. In developed countries such as the USA and UK, children commonly sustain ocular injury during play and sports such as soccer, badminton, archery and darts.^26^ In contrast, for children from developing countries or low socioeconomic status, ocular trauma often occurs during play with non-traditional objects like broomsticks or khebab sticks used as missiles.^29^ Broomstick injuries are particularly an important cause of monocular blindness as was seen in the study by Essuman et al^28^ who found that 50% of children who presented with broomstick injuries became blind in the affected eye. Ukponmwan et al^29^ reported that three-quarters of the children presenting with broomstick injuries were blind (vision worse than 3/60) at their last follow-up. Poor visual outcomes in broomstick injuries are attributable to broomsticks serving as reservoirs for multiple pathogenic bacteria and fungi, leading to fulminating ocular infections such as endophthalmitis.^29^ In this current study, 10 children (9.7%) sustained broomstick injuries, with all 10 being males. Pencil or pen tip injury was another important cause of ocular trauma in our study, accounting for 7.7% of cases. One study participant had an IOFB. The child sustained ocular trauma a month prior to presentation and was managed conservatively at a nearby health facility. He, however, reported recurrent pain and redness in the affected eye. On examination, a sealed corneal perforation plugged with iris was noted and a dark object was observed in the anterior chamber which was later found to be a lead pencil tip, which was surgically removed. Tabatabaei et al^31^ In their study from Iran, they reported 36 cases of ocular trauma caused by writing objects such as pencils and pens over a 12-month period.

Globe trauma occurred in over three-quarters of our patients. This finding corroborates what was reported in the UK POTS 3, which showed that 76.7% of ocular trauma involved the globe.^8^ Based on the BETT classification, the current study demonstrated that most of the study participants had open globe injury, and a statistically significant difference was observed between the proportion of OGIs and CGIs (p=0.004). This is also consistent with the UK POTS 3 study, which also reported the majority (60.1%) of children presented with open globe injury.^8^ It further corroborates findings by Madan et al^11^ who reported 63.9% of ocular trauma to be open globe injuries from their study in India. These findings, however, differ from those of an Australian study by Liu et al^16^ who reported that most cases of paediatric ocular trauma were CGIs (68.5%). It also differs from studies by Merca et al(Philippines),^6^ Puodžiuvienė et a(Lithuania)^7^ and Al-Mahdi et al(Qatar)^32^ who also reported that CGI accounted for the majority of cases (54.7%, 53.4%, and 59.4% respectively) in their study cohorts. The difference in these findings could be explained by the differences in the sampled populations. This current study, as well as the study from the UK, included children under the age of 16 years, while the studies by Merca et al^6^ and Puodžiuvienė et al^7^ included children above the age of 16 years. It has been demonstrated that OGIs are common in younger children, while blunt trauma occurs more frequently in older children.^6^,^12^,^16^ Younger children are more likely to get injured accidentally by sharp objects, while older children are more likely to be injured during fist fights or sports.^7^,^16^ In this study, more older children (11-15 years) were injured due to fist fights, and the difference was statistically significant (p = 0.006). Almost all the children in our study with OGI received surgical treatment except 2, due to failure of consent by their parents. One parent cited religious belief as the reason. The child was admitted for surgical management, but the parents absconded, only to report after 2 weeks. Unfortunately, the eye had become phthisical.

At presentation, only 27.6% of children had normal vision (6/12 or better), and at 6-week follow-up, children with normal vision increased from 27.6% at baseline to 47.4%. This is, however, lower than visual outcomes reported by Liu et a^16^ in a paediatric ocular trauma study in Australia and another study conducted in the Philippines.^6^ The proportions of children achieving final VA of 6/12(0.3 LogMAR) or better in the studies reported by Liu et al^16^ and Merca et al^6^, were 69% and 76.2% respectively. The proportion of children achieving final VA ≥ 6/12(0.3 LogMAR) in this current study was also lower than what was reported in a study from Canada where 56.5% achieved final VA ≥ 6/12(0.3 LogMAR).^19^ The difference could be due to the fact that in this current study, OGIs were the predominant type of injury, in contrast to the studies from Australia and Philippines. Closed globe injuries are generally reported to have better visual outcomes compared to open globe injuries.^7^,^15^ Another explanation for the lower proportion of children achieving VA ≥ 6/12(0.3 LogMAR) could be due to the shorter follow-up period for this study, unlike studies from Australia and the Philippines with follow-up exceeding 2months. It is possible that the best corrected VA will continue to improve in study participants following refraction and spectacle prescription as well as amblyopia management.

Our study found that children with open-globe injuries had poorer visual outcomes than those with closed-globe injuries. Children with closed-globe trauma were 11 times more likely to have favourable outcomes. This could be because presenting visual acuities were generally poorer in children with OGIs. Other studies comparing visual outcomes from open and closed globe injuries have also affirmed the same.^6^,^12^,^33^,^34^ OGIs are likely to have more tissue damage and are more likely to require surgical intervention.^35^ Patients with OGIs also have an additional risk of secondary microbial infections, especially in cases such as broomstick injury, leading to endophthalmitis, which is a devastating complication and leads to poorer visual outcomes.^36^

The use of unorthodox treatments, such as the application of breastmilk and holy water, and washing of the face with water, occurred in 7.8% of children. One child with penetrating ocular trauma had “Holy water” applied to the eye for 3 days prior to presentation. A second child, also with penetrating eye injury, had breast milk applied to the eye for 4 days prior to presentation. In the latter case, parents refused surgical treatment and reported back after 2 weeks when the eye had become phthisical, and vision was NPL. Univariate and multivariate analyses, however, did not show any significant association between the type of pre-hospital management and visual outcome.

Only about a third of the children presented within 24 hours after injury. This is comparable to a study conducted in Nigeria by Kyari et al^18^ who found that the majority of children with ocular trauma presented between 2 and 6 days after injury. This is in contrast to a study from China, where the majority of children (54%) presented within 24 hours after injury.^21^ Delayed presentation and/or treatment of ocular trauma can lead to complications such as endophthalmitis, which worsen the visual prognosis.^37^ Some studies, however, have not found delay in presentation or treatment as a prognostic indicator of visual outcome.^25^ For instance, Grieshaber et al^25^ observed that delay in presentation was not of prognostic value. In that same study, however, all the children who developed endophthalmitis presented more than 5 days after injury.^25^ Delayed presentation may stem from various factors such as financial constraint, lack of appreciation of dangers associated with ocular trauma, failure of children to report trauma to parents on time and difficulty in accessing healthcare facilities in some instances.^1^,^11^,^24^ In our study, univariate analysis showed that children with ocular trauma who present early are 3 times more likely to have favourable visual outcomes compared to those who present late. There is considerable evidence to support the claim that the distance between the location of an eye injury and the hospital is a major factor in delayed presentation, especially in underserved or rural areas with limited access to medical facilities.^38^ Unfortunately, this link was not examined in our study.

Both univariate and multivariate analyses showed that children injured by organic objects had a worse visual outcome. Organic objects such as sticks, broomsticks and stones are usually contaminated by various microorganisms, which predispose children with OGI to endophthalmitis. In India, a study on post-traumatic endophthalmitis in children found broomstick injury was the commonest cause.^39^ In a study on broomstick injuries by Ukponmwan et al^29^ in Nigeria, 75% of children were blind in the affected eye at the last follow-up. Essuman et al^28^ In Ghana, it was also reported that 50% of ocular trauma resulting from broomstick injuries resulted in blindness. In this study, sticks were the second most common cause of injury, followed by stones and broomstick injuries.

The POTS is a useful tool that has been validated by many studies to help in prognosticating visual outcomes post OGIs.^40^,^41^ In this study, there was a strong and significant negative correlation between paediatric ocular trauma scores and final visual acuity in LogMAR. Thus, lower scores were associated with poorer visual outcomes, and vice versa. This tool is therefore very useful for counselling guardians and children with open globe injuries regarding expected visual outcomes after intervention.

Despite efforts such as collecting contact numbers and appointment reminders, five children were lost to follow-up, with some unreachable by phone. This number was below the projected 15% dropout rate. The study followed participants for 6 weeks; further improvement in visual acuity may occur over time.

At the initial presentation, the visual acuities of 5 children could not be assessed due to poor cooperation. At 6 weeks, one child did not allow any form of visual acuity testing. This child was excluded from the final analysis of visual outcomes.

Despite these few challenges, this study has provided important data on the epidemiology of paediatric ocular trauma and has identified factors influencing visual outcome in a large paediatric cohort in Ghana. This would provide an important basis to inform public education on this problem, thereby contributing to the prevention of childhood blindness and visual impairment in Ghana.

## Conclusion

Most children with ocular trauma were boys aged 6-10 years, highlighting the need for targeted school eye health programs. Globe injuries made up three-quarters of cases, mainly open globe injuries. Over one-third remained blind in the affected eye after 6 weeks, though overall visual acuity improved significantly by the last follow-up. OGIs and injuries from organic objects led to worse outcomes. Improved public health education and adult supervision are necessary to prevent these injuries.

## Figures and Tables

**Table 1:** Paediatric ocular trauma score for open globe injuries

Variable	Raw points
***Initial VA***	
**NPL**	10
**PL/HM**	20
**Counting fingers**	30
**0.1-0.5**	40
**0.6-1.0**	50
***Age of paediatric patient***	
**0-5**	10
**6-10**	15
**11-15**	25
***Wound location***	
**Zone 1**	25
**Zone 2**	15
**Zone 3**	10
***Concomitant eye pathologies***	
**Iris prolapse**	-5
**Hyphaema**	-5
**Organic/unclean injury**	-5
**Delay of surgery (48 h)**	-5
**Traumatic cataract**	-10
**Vitreous haemorrhage**	-20
**Retinal detachment**	-20
**Endophthalmitis**	-30

**Table 7:** Multivariate analysis of factors influencing visual outcomes post ocular trauma in children at the Lions International Eye Centre, Korle Bu Teaching Hospital

Risk factor	Odds ratio	95% Confidence interval	P-value
**Age group**			
**<10 years** **10-15 years**	0.8 Ref. cat.	0.2-3.0 -	0.713
**Sex:**			
**Male Female**	0.5 Ref. cat.	0.1-1.5	0.203
**Time of presentation:**			
**Early** **Late**	0.4 Ref. cat.	0.1-1.1 -	0.068
**Type of injury:**			
**Closed** **Lid laceration** **Open**	8.2 2.3 Ref. cat.	1.4-46.7 0.3-18.2 -	0.018* 0.420
**Baseline VA**			
**Favourable** **Unfavourable**	0.0 Ref. cat.	0.0	0.991
**Cause of injury:**			
**Inorganic** **Organic**	3.7 Ref. cat.	1.3-10.8	0.016*

* Significant values. Ref. cat. = reference category; VA= visual acuity
